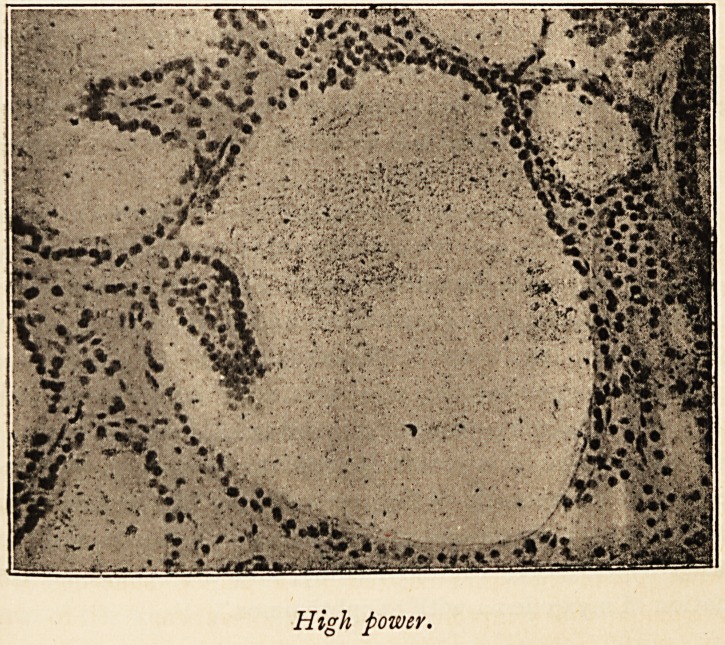# Notes on a Case of Exophthalmic Goitre

**Published:** 1896-03

**Authors:** F. H. Edgeworth

**Affiliations:** Assistant-Physician to the Bristol Royal Infirmary


### NOTES ON A CASE OF EXOPHTHALMIC GOITRE.

BY

F. H. Edgeworth, M.B., B.A. Cantab., B.Sc. Lond.,
*Assistant-Physician to the Bristol Royal Infirmary.*

---

Jane B., aged 34, was admitted to the Royal Infirmary on June 17th, 1895, suffering from well-marked exophthalmic goitre.

The family history was good, and the patient had been quite healthy until about five years previously, when she nursed her husband through a long and fatal illness, and subsequently had great difficulty in finding work to support two children and herself.

About three months after her husband's death she first noticed the symptoms of the disease. She managed, though with difficulty, to earn her living during the first four years, but in the course of the last six months found herself gradually incapacitated by reason of increasing weakness and palpitation.

On admission it was found that the ordinary symptoms of Graves's disease were present. These need be but briefly mentioned: there was considerable exophthalmos with Von Graefe's and Stellwag's signs; all three lobes of the thyroid gland were enlarged, forming a firm but elastic tumour. A systolic thrill could be felt and heard over the superior and inferior thyroid arteries. The heart was slightly hypertrophied, and the cardiac and pulse-rate varied from 120 to 130 per minute. The vascular excitability was extreme: a slight emotion would make the patient flush over head, neck, and chest, and send the pulse up to 150 per minute. There was slight muscular tremor. The patient was a good deal emaciated. The temperature varied between 100° and 101° F.

The patient was kept in bed and belladonna administered in increasing doses. Under this treatment the pulse-frequency gradually declined, and at the end of a month was, on an average, 100 per minute; the temperature became normal, and the patient thought herself better. It was found, however, that even a few hours out of bed sent the temperature up to 100° and the pulse-rate to 120. In short, no permanent improvement was effected, and the patient was, at the end of six weeks, as unable to follow her employment as before.

I therefore asked Mr. Harsant to consider the question of partial excision of the thyroid. He advised operation, and at the end of July removed the right lateral lobe of the gland. The result, unfortunately, was disastrous—though the operation lasted a very short time and the bleeding was slight, yet in a little over thirty hours the patient died. The temperature gradually rose to 103°, the pulse-rate increased to 130 per minute, there was some delirium, and death took place from heart-failure.

The only macroscopic *post-mortem* changes found were the enlargement of the left lateral and median lobes of the thyroid gland, and hypertrophy and dilatation of the heart. The wound looked quite healthy. Microscopic examination of sections of the thyroid show the changes described by Greenfield. There is no increased vascularity of the gland, though the superficial veins were enlarged. The epithelial cells lining the vesicles are changed from a cubical to a columnar form, and there are papillary projections of the epithelial cells into the vesicles. The series of changes can be easily traced; some vesicles are lined by cubical cells only, in others the cells in part of the circumference have become columnar, and in other vesicles again these columnar cells have proliferated to form the papillary projections. The alterations of structure evidently begin locally in the circuit of cells lining a vesicle, and do not affect the whole circumference at once. The production of duct-like spaces lined by cubical cells and the development of connective tissue, described by Greenfield, are not obvious.

These anatomical changes certainly suggest a heightened activity of the gland. Further than this they do not take us—to ascertain its nature we must turn to other evidence. If thyroid gland be administered to a person suffering from myxœdema, the symptoms gradually disappear. If to such

*Low power.*

*High power.*

a patient or to a healthy person overdoses of the gland be given, many of the symptoms of Graves's disease, *e.g.* tachycardia, heightened temperature, wasting, occur. But one characteristic symptom, the exophthalmos, is not so produced.

It may be inferred that in Graves's disease the disordered metabolism results in the production of two different substances —(1) an excess of normal thyroid secretion, and (2) a substance which causes the exophthalmos and other eye symptoms. The effects of cocaine on the eye have been brought forward by Edmunds in support of the theory that a substance of somewhat similar properties may possibly be produced by the thyroid. We may also suppose that there is no necessary quantitative relation between the secretion of these two substances; for, as is well known, there may be tachycardia without, or with but little, exophthalmos, and conversely.

Further, whilst the degree of exophthalmos is, so to speak, an isolated phenomenon, the amount of cardiac disturbance is, apart from the question of subsequent heart-failure, in more or less close relation to the general bodily disturbance.

Again, in some cases the disturbance of thyroid metabolism may be supposed to be such that whilst there is a diminution or arrest of the normal secretion, that of the exophthalmos producing substance still goes on. This would result in a combination of myxœdema and exophthalmos. Such conditions have actually been observed, especially as sequels of Graves's disease.

Other combinations of the symptoms of Graves's disease and exophthalmos are, according to this theory, not possible, and, as far as I have been able to read, have not been recorded. Thus we do not find myxœdema and tachycardia. If we suppose, then, that the "perverted function" of the thyroid, spoken of by some authors, is of this dual nature—a secretion of an exophthalmos producing substance, and an excess of normal secretion—it is possible to explain the non-parallelism of the various phenomena of Graves's disease, the likenesses and differences between the symptoms of Graves's disease and those caused by excessive thyroid feeding, and the occasional combination of myxœdema with exophthalmos.

The course taken by the "internal" secretion of the thyroid gland—the failure of which is the proximate cause of myxœdema —merits discussion. It may be inferred from analogy that this secretion does not first pass into the thyroid vesicles, but that it passes directly from the external surface of the lining cells into the lymphatics and thence into the blood. For in the case of the pancreas it is pretty well established that whilst the digestive juices enter the duct, the "internal" pancreatic secretion—failure of which is the cause of some cases of glycosuria—passes at once into surrounding lymphatics and so into the blood. And the thyroid was originally a gland pouring a secretion into the anterior end of the alimentary canal.

The ancestral glandular, colloid-producing, function of the thyroid has a tendency to disappear. Thus a part of the gland consists of epithelial cells not arranged into vesicles, but merely aggregated together and producing no "external" secretion. And in some animals, *e.g.* the rabbit, where these cells are separated off as an accessory thyroid, it has been shown by Gley and by Edmunds that myxœdema does not occur unless the accessory as well as the main thyroid glands are excised. In such accessory thyroids the, probably secondary, function of producing an "internal" secretion alone persists.

From an anatomical point of view, then, the terms "internal" and "external" are misapplied: whilst an "external" secretion is one which is poured into a duct or upon a free surface, an "internal" one passes into the blood and influences tissue metabolism: it is from a physiological point of view that the use of the terms is justified.

The influence of the "internal" thyroid secretion on nitrogenous and non-nitrogenous metabolism may here be touched upon. It has been shown by Ord and White that if thyroid gland be administered to a person suffering from myxœdema, the body weight diminishes, and there is a large increase in the nitrogenous excreta almost entirely in the form of urea, whilst correspondingly the quantity of urine is increased. Evidently there is an increase in the rate of nitrogenous metabolism. Such increase is, in large part at any rate, the cause of the wasting which occurs in Graves's disease.

It may here be remarked, though it is a side issue, that thyroid gland may hence be given as a diuretic. Thus, to a non-myxœdematous woman, aged 41, suffering from ascites due to chronic peritonitis, where the average amount of urine was 20 oz. during a period of 14 days, the administration of 15 grs. of dried thyroid gland daily caused during the next 14 days an average secretion of 36 oz. urine. Since, however, this diuresis is due mainly, if not entirely, to the increase in the urea, it is at the expense of the tissues that such an action takes place. It is better to give urea by the mouth.

Very occasionally glycosuria occurs as a complication of Graves's disease, and it is of some interest to enquire whether there is any causal connection between the two phenomena, whether the "internal" secretion of the thyroid has any marked action on non-nitrogenous, as well as on nitrogenous, metabolism. The diminution of subcutaneous fat in a person suffering from Graves's disease, and the effects of administration of thyroid in cases of obesity, suggest that it has.

To further test the question, thyroid gland was given to a boy, aged 15, suffering from diabetes; the result was an increase in the amount of sugar excreted when large quantities of thyroid were administered, as shown in the following table:

| | Average daily quantity of urine. oz. | Average daily quantity of sugar. grs. |
|---|---|---|
| 1st period of 8 days, diabetic diet, no opium, no thyroid | 76 | 1600 |
| 2nd " 14 " " " opium 1 gr., " | 57 | 1110 |
| 3rd " 15 " " " " " 5 grs. dried thyroid | 55 | 930 |
| 4th " 6 " " " " " 10 " " | 81 | 2250 |
| 5th " 6 " " " " " no thyroid | 68 | 920 |

Thus, it would seem that whilst in ordinary cases of Graves's disease and in cases where thyroid is administered the increase in non-nitrogenous metabolism does not result in the production of sugar, yet its occasional occurrence is not altogether obscure.

The results above noted show the need of the greatest caution in giving thyroid gland as any part of the treatment of obesity, in which slight glycosuria is not so rarely present.

Some light has recently been thrown on the obscure connection between the "perverted function" of the thyroid in

Graves's disease and the well-known etiology of the complaint. As Gowers says, " No immediate cause is so frequent as depressing emotion—sudden terror or prolonged distress." For it has been shown by Berkeley that the thyroid gland has a liberal supply of nerve fibres, which, entering alongside of the blood-vessels, form anastomoses round the vesicles and send off numerous fine branches which end in small knobs in direct contact with the outside of, or between, the epithelial cells. There is thus a direct connection between the secreting cells and the central nervous system. And just as the central nervous system controls and regulates the production of "external" glandular secretions, *e.g.* the saliva, so is it probable that it also controls and regulates the production of "internal" glandular secretions.

In conclusion, a practical point may be briefly discussed. Are those of us who accept the thyroid view of Graves's disease justified in recommending operation as a mode of treatment? In slighter cases, rest in bed and the administration of belladonna—the drug which of all others does most good, perhaps by inhibiting secretion—suffice to bring about a cure. In more severe cases, excision of part of the gland will, if the patient survive the first few days, produce a very great improvement or recovery. But it is precisely in these cases that there is considerable risk to life—a risk which it is not in the surgeon's power to diminish—and such a result as that recorded above must make one hesitate a little in proposing operation, even when the disease is well marked.

I have much pleasure in thanking Mr. James Taylor for the photo-micrographs.

## Figures and Tables

**Figure f1:**
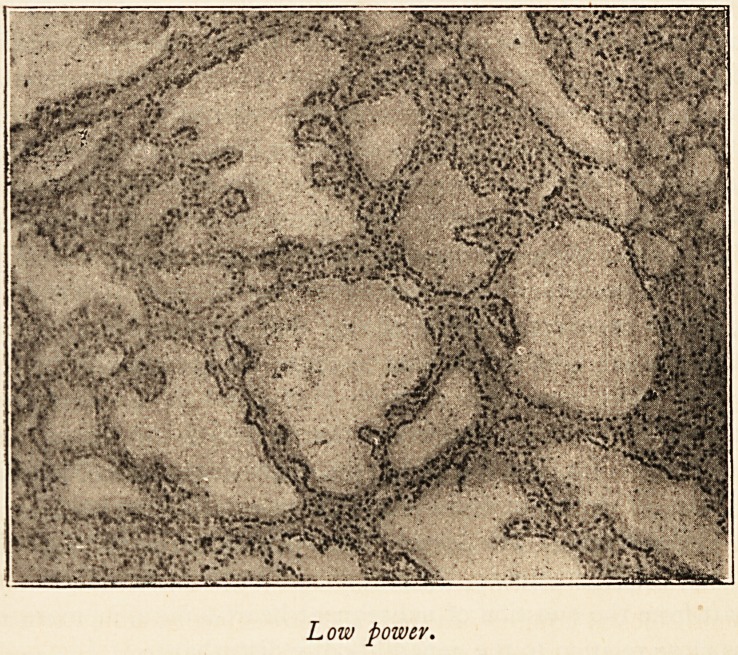


**Figure f2:**